# Neurosarcoidosis Presentation as Adipsic Diabetes Insipidus Secondary to a Pituitary Stalk Lesion and Association with Anti-NMDA Receptor Antibodies

**DOI:** 10.1155/2020/7956350

**Published:** 2020-06-25

**Authors:** Jose Gabriel Solis, Arturo Olascoaga Lugo, Marco Antonio Rodríguez Florido, Bayron Alexander Sandoval Bonilla, Jose Malagón Rangel

**Affiliations:** ^1^Department of Internal Medicine, Hospital de Especialidades “Dr. Bernardo Sepúlveda Gutiérrez”, Centro Médico Nacional Siglo XXI, Instituto Mexicano del Seguro Social, Ciudad de México, Mexico; ^2^Department of Pathology, Hospital de Pediatría Centro Médico Nacional Siglo XXI, Instituto Mexicano del Seguro Social, Ciudad de México, Mexico; ^3^Department of Neurosurgery, Hospital de Especialidades “Dr. Bernardo Sepúlveda Gutiérrez”, Centro Médico Nacional Siglo XXI, Instituto Mexicano del Seguro Social, Ciudad de México, Mexico

## Abstract

Sarcoidosis is a multisystemic inflammatory disease of unknown cause. It is characterized by the presence of noncaseating granuloma on a biopsy specimen. Clinical presentation varies across case report series with myriad of symptoms ranging from fever, respiratory symptoms, and skin lesions, or atypical symptoms like heart block or neurological symptoms. Hence, we report the case of a 22-year-old woman with encephalitis, a pituitary mass, and adipsic diabetes insipidus. The diagnostic approach did not end on the biopsy of the lesion, which reported noncaseating granulomas; on the contrary, it was the beginning of a path to exclude other causes of the central nervous system granulomas that ended with the diagnosis of the isolated central nervous system sarcoidosis. Also, we report the first proven association between anti-NMDA receptor antibodies and sarcoidosis.

## 1. Introduction

Sarcoidosis is a multisystemic inflammatory disease of unknown cause, whose primary feature is the presence of noncaseating granulomas [1]. It has an incidence of less than 10 cases per 100,000 people, with a predilection to the ages of 20–40 years. There is evidence of lung involvement in 95% of the cases [2]; however, neurosarcoidosis is present only in 5–15% of the cases, of which in 50% is the initial manifestation. Isolated neurosarcoidosis is very rare, and up to a third of the patients will develop systemic features in the future [3].

We report the case of a patient who presented with adipsic diabetes insipidus secondary to a pituitary stalk lesion, with the final diagnosis of neurosarcoidosis. Also, the patient had neuropsychiatric symptoms and anti-NMDA antibodies detected in cerebrospinal fluid, an association that has never been reported.

## 2. Case Presentation

A 22-year-old woman, resident of Acapulco, Mexico, presented to our unit with a two-month clinical picture of headache, incoherent language, aggressive behavior, inappropriate conducts of disinhibition, and visual-auditory hallucinations. The previous day, the patient had gradually developed a diminished state of awareness. Her medical history was unremarkable. She had two uncomplicated previous pregnancies. Her mother denied any animal contact, recent traveling, ingestion of drugs, or exposure to toxics. She referred a lack of water and food intake in the previous days.

On physical examination, her blood pressure was normal, and she was tachycardic with a weak pulse, dry mucous membranes, and prolonged capillary refill time. In the neurological sphere, she was disoriented and presented retrograde amnesia, impairment in judgment, and loss of abstract thinking. Her chest X-ray and head computed tomography (CT) were normal. Laboratory results revealed severe hypernatremia (Na 180 mEq/L) and acute kidney injury (AKI) (measured creatinine 2 mg/dl–baseline creatinine 0.6 mg/dl). Serum electrolytes, liver tests, blood count, urinalysis, and toxicologic screen were normal. Her spot urinary electrolytes showed a urinary sodium of 20 mEq/L, with a fractional excretion of sodium (Fe_Na_) of 0.5% suggesting hypovolemia. Her calculated water deficit was of 7.5 liters. However, her calculated electrolyte-free water excretion was of 805 ml within a 24-hour urinary volume of 1,000 ml, a value that was inappropriately elevated.

**Figure 1:**
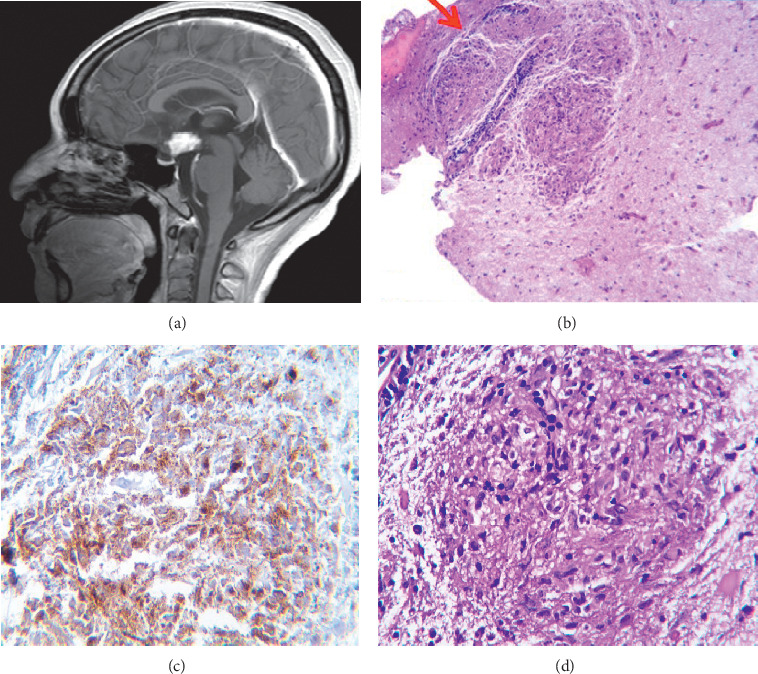
(a) MRI T1-weighted sequence showing a pituitary stalk lesion with marked reinforcement and chiasmatic and hypothalamic extension. (b) Perivascular granulomas HE 10x. (c) CD 68 positive in epithelioid cells. IHQ 40x. (d) Granuloma composed by epithelioid cells and reactive gliosis at the periphery. HE 40x. MRI, magnetic resonance imaging. IHQ, immunohistochemistry. HE, hematoxylin and eosin.

We initiated treatment with enteral administration of free water and hypotonic intravenous solutions for restitution of intravascular volume. During her hospital stay, the patient presented a partial clinical response, with optimization of volemia and resolution of (AKI). A new electrolyte profile showed persistence of hypernatremia (Na 157 mEq/L) and mild hypokalemia (K 3.2 mEq/L). She had a urinary sodium level of 8 mEq/L, with a calculated urine osmolarity of 85 mEq/L, in the context of a 24-hour urinary volume of 4 liters and electrolyte-free water excretion of 3.3 liters. The documented aqueous polyuria and hypernatremia suggested diabetes insipidus. The water deprivation test, that is normally the next step in the diagnosis, was contraindicated, so we ordered a brain magnetic resonance imaging (MRI), which showed a pituitary stalk lesion with chiasmatic and hypothalamic extension (Figure 1(a)).

**Figure 2:**
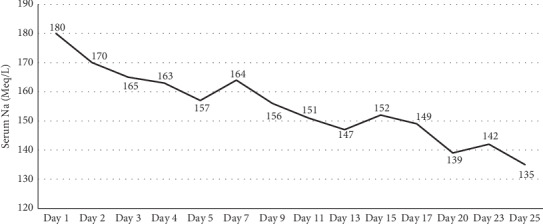
Graphical representation of serum sodium levels behavior during hospitalization. Desmopressin was administered from day 5 according to requirements.

Treatment with desmopressin was initiated, with progressive normalization of her sodium level, urinary volume, and volemia status (Table 1) (Figure 2). The hormonal profile was compatible with panhypopituitarism, so we initiated treatment with thyroid and glucocorticoid hormones. The cerebrospinal fluid (CSF) analysis showed an elevated protein level of 75 mg/dl, normal glucose, a cell count of zero, and negative Gram and India ink stains. An electroencephalogram documented diffuse cerebral dysfunction without the epileptiform activity. Her blood, urine, CSF, and bone marrow cultures were all negative. A CSF polymerase chain reaction (PCR) for the detection of viral agents and *Mycobacterium tuberculosis* was negative. The determination of anti-N-methyl-D-aspartate (NMDA) receptor antibodies in CSF was positive. Diverse imaging studies such as thoraco-abdominopelvic CT, endovaginal ultrasound, and whole-body scintigraphy showed no significant abnormalities.

Considering the characteristic clinical behavior of the patient, we decided to order a stereotactic biopsy of the pituitary mass. The pathology report revealed noncaseating granulomatous inflammation and gliosis (Figures 1(b)–1(d)). The periodic acid Schiff stain (PAS), Ziehl–Neelsen stain, and Grocott stain of the tissue were all negative. Immunohistochemical markers CD1a and langerin to rule out Langerhans cell histiocytosis were negative. Serological tests for Epstein–Barr, hepatitis B, hepatitis C, cytomegalovirus, toxoplasma, and *Brucella spp* were all negative. A PCR for the detection of *Rickettsia spp*, *Ehrlichia spp*, *Anaplasma spp*, and *Borrelia burgdorferi* were also negative. A nested PCR in biopsy tissue for the detection of *Mycobacterium tuberculosis* was also negative. The angiotensin converting enzyme serum level was in the normal range.

Based on the pathology findings and the 2018 consensus diagnostic criteria [4], we concluded the final diagnosis of isolated neurosarcoidosis. She received treatment with prednisone and showed significant improvement of the neuropsychiatric symptoms. The patient was discharged from our unit to continue ambulatory treatment; however, she was lost at the follow-up.

## 3. Discussion

Neurosarcoidosis can infiltrate practically any structure of the central nervous system. The most frequent manifestation is cranial neuropathy; however, aseptic meningitis, cauda equina syndrome, diffuse encephalopathy, focal neurological symptoms, hypothalamic and pituitary disfunction, intracranial hypertension, peripheral neuropathy, and vascular syndromes have all been described [5].

We report the case of a 22-year-old female patient with a pituitary granulomatous infiltrative lesion. This is the most frequent and characteristic form of the disease and is present in 15–25% of the cases. This can produce symptoms related to the mass effect or endocrine dysfunction [3, 6]. The patient had multiple endocrine manifestations of the disease, which are reported with the following frequencies: hypogonadotropic hypogonadism (38.5%), diabetes insipidus (37%), amenorrhea (29%, 58.7% of women), panhypopituitarism (23%), visual defects (22%), headache (7.7%), and psychoaffective changes (7.7%) [7]. Hyperprolactinemia has been considered as a predictor of hypothalamic involvement [8].

Polyuria-polydipsia syndrome in these patients is usually multifactorial and can be secondary to central diabetes insipidus, primary polydipsia, nephrogenic diabetes insipidus secondary to hypercalcemia, or less frequently dysfunction of the thirst control mechanism [9]. The latter is a very rare type of diabetes insipidus, in which there is a deficient or absent thirst response to hypernatremia and is associated with more morbidity and mortality. Descriptions of neurosarcoidosis as a cause of this variety of diabetes insipidus has been anecdotal [10]. Central diabetes insipidus in sarcoidosis has been described in case reports or series of cases [11, 12]; however, this as a form of presentation is extremely rare and has only been reported in a 10-year-old girl [13]. The only published follow-up study including patients with sarcoidosis and diabetes insipidus found a clinical profile very similar to that of our patient. Those patients started at ages between 18 and 33 years and had associated neurological symptoms and endocrine defects. Peculiarly, the polyuria in these patients did not respond to treatment with steroids [12].

The initial clinical features of our patient were associated with hypovolemia and severe hypernatremia. We can deduce that these were masking the polyuria expected in diabetes insipidus and were exacerbated by the dysfunction of the thirst mechanism and neuropsychiatric manifestations. In the context of neurosarcoidosis, the diagnosis of diabetes insipidus can be hard because it can be masked by hypovolemia, adrenal insufficiency [14], or hypothyroidism [15], which are contraindications to perform a water deprivation test.

Pituitary stalk lesions have a broad differential diagnosis. Turcu et al. published a retrospective description of patients with pituitary stalk lesions conducted at the Mayo Clinic. In this study, neurosarcoidosis was the most common inflammatory etiology, and it was found in 11 of 92 patients [16]. The most frequent MRI finding was a thickened pituitary stalk [16]. Other possible findings include an intrasellar well-defined lesion with hyperintense behavior in the T1 sequence, which can have suprasellar extension, a cystic component, or leptomeningeal reinforcement, although the findings can be nonspecific [17]. In the CSF analysis, an elevated protein level is the most frequent finding present in 63% of the patients. An elevated angiotensin converting enzyme is relatively rare, only observed in 35% of the patients, which is the reason we did not consider its normal value relevant in our case [18].

Diagnostic criteria were proposed by the neurosarcoidosis consortium consensus group in 2018, although they have not been prospectively validated [4]. Our patient fulfilled the diagnostic criteria for definite neurosarcoidosis, which includes a clinical presentation and diagnostic evaluation compatible with the disease, the central nervous system pathology consistent with neurosarcoidosis, and rigorous exclusion of other causes [4].

An important clinical feature in our patient was the presence of psychiatric and encephalopathic symptoms. These types of symptoms in neurosarcoidosis are considered secondary to granulomatous inflammation, although this has not been confirmed. Remarkably, our patient had anti-NMDA receptor antibodies detected in CSF, an association that has never been reported in medical literature. Limbic encephalitis secondary to neurosarcoidosis was present in a case report, in which it was considered secondary to direct infiltration of the disease; however, in this case, anti-NMDA receptor antibodies were not measured [19]. This could imply that there is an autoimmune component of neuropsychiatric symptoms in patients with neurosarcoidosis. There is a case report of anti-NMDA receptor encephalitis in a 3-year-old boy secondary to microdeletion of the chromosome 6p21.32 that includes the HLA-DPB1 y HLADPB2 genes [20], which have been also associated with the development of sarcoidosis [21]. This could also be related to a B cell-mediated encephalopathy [22].

In conclusion, we report the case of a very rare presentation of neurosarcoidosis whose almost unique clinical features have only been described in case reports. The etiologic diagnosis of pituitary stalk lesions is difficult, which highlights the need of performing a biopsy and a histopathologic diagnosis, especially in atypical cases. This has fundamental therapeutic implications. To our knowledge, this is the first report of documented anti-NMDA antibodies in a definite case of neurosarcoidosis. This may be a reason for future research.

## Figures and Tables

**Table 1:** Serum and urinary sodium levels, osmolarity, urinary volume, and calculated electrolyte-free water excretion during hospitalization. Desmopressin was administered from day 5 according to requirements.

	Day 1	Day 2	Day 3	Day 4	Day 5	Day 7	Day 9	Day 11	Day 13	Day 15	Day 17	Day 20	Day 23	Day 25
Serum Na (mEq/L)	180	170	165	163	157	164	156	151	147	152	149	139	142	135
Urinary Na (mEq/L)	20	17	5	9	8	33	17	113	50	170	127	29	65	188
Serum osm (mOsm/l)	366	345	336	331	326	337	317	307	299	308	306	284	272	276
Urinary osm (mOsm/l)	219	342	380	225	85	574	339	580	305	512	750	144	290	474
Urinary volume (lts)	1	1.25	2	3	4	1.85	2.5	1.8	1.5	1.9	2	2.8	2	1.5
EFWE (lts)	0.805	0.888	1.468	2.52	3.3	−0.56	1.21	−0.239	0.613	−0.42	−1	2	1.09	−1.16

Na, sodium; Osm, osmolarity; EFWE, electrolyte-free water excretion.
